# Exploiting transcriptome data for the development and characterization of gene-based SSR markers related to cold tolerance in oil palm (*Elaeis guineensis*)

**DOI:** 10.1186/s12870-014-0384-2

**Published:** 2014-12-19

**Authors:** Yong Xiao, Lixia Zhou, Wei Xia, Annaliese S Mason, Yaodong Yang, Zilong Ma, Ming Peng

**Affiliations:** Hainan Key Laboratory of Tropical Oil Crops Biology/Coconut Research Institute, Chinese Academy of Tropical Agricultural Sciences, Wenchang, Hainan 571339 P.R. China; Institute of Tropical Bioscience and Biotechnology, Chinese Academy of Tropical Agricultural Science, Haikou, Hainan 571101 P. R. China; School of Agriculture and Food Sciences and Centre for Integrative Legume Research, the University of Queensland, 4072 Brisbane, Australia

## Abstract

**Background:**

The oil palm (*Elaeis guineensis*, 2n = 32) has the highest oil yield of any crop species, as well as comprising the richest dietary source of provitamin A. For the tropical species, the best mean growth temperature is about 27°C, with a minimal growth temperature of 15°C. Hence, the plantation area is limited into the geographical ranges of 10°N to 10°S. Enhancing cold tolerance capability will increase the total cultivation area and subsequently oil productivity of this tropical species. Developing molecular markers related to cold tolerance would be helpful for molecular breeding of cold tolerant *Elaeis guineensis*.

**Results:**

In total, 5791 gene-based SSRs were identified in 51,452 expressed sequences from *Elaeis guineensis* transcriptome data: approximately one SSR was detected per 10 expressed sequences. Of these 5791 gene-based SSRs, 916 were derived from expressed sequences up- or down-regulated at least two-fold in response to cold stress. A total of 182 polymorphic markers were developed and characterized from 442 primer pairs flanking these cold-responsive SSR repeats. The polymorphic information content (PIC) of these polymorphic SSR markers across 24 lines of *Elaeis guineensis* varied from 0.08 to 0.65 (mean = 0.31 ± 0.12). Using *in-silico* mapping, 137 (75.3%) of the 182 polymorphic SSR markers were located onto the 16 *Elaeis guineensis* chromosomes. Total coverage of 473 Mbp was achieved, with an average physical distance of 3.4 Mbp between adjacent markers (range 96 bp - 20.8 Mbp). Meanwhile, Comparative analysis of transcriptome under cold stress revealed that one *ICE1* putative ortholog, five *CBF* putative orthologs, 19 *NAC* transcription factors and four cold-induced orhologs were up-regulated at least two fold in response to cold stress. Interestingly, 5′ untranslated region of both Unigene21287 (*ICE1*) and CL2628.Contig1 (*NAC*) both contained an SSR markers.

**Conclusions:**

In the present study, a series of SSR markers were developed based on sequences differentially expressed in response to cold stress. These EST-SSR markers would be particularly useful for gene mapping and population structure analysis in *Elaeis guineensis*. Meanwhile, the EST-SSR loci were inducible expressed in response to low temperature, which may have potential application in identifying trait-associated markers in oil palm in the future.

**Electronic supplementary material:**

The online version of this article (doi:10.1186/s12870-014-0384-2) contains supplementary material, which is available to authorized users.

## Background

Oil palm (*Elaeis guineensis* Jacq., 2n = 32), belonging to the genus *Elaeis* in the monocotyledonous family Arecaceae (Palmaceae), is an important tropical oil crop. The genus *Elaeis* consists of two different species, *Elaeis guineensis* (African oil palm) and *Elaeis oleifera* (American oil palm) [1]. *Elaeis guineensis* is currently commercially cultivated for palm oil production in the tropics, particularly in Indonesia and Malaysia. Some efforts have been made to introduce African oil palm into subtropical regions in regional trial plantation, including in the Hainan province located in the southern China. However, winter temperatures in these regions are generally lower than 20°C (and can even low than 10°C), which resulted in slowing of flower bud differentiation and fruit development, subsequently severely affecting the oil palm fruit productivity. Hence, enhancing cold tolerance in this tropical species is a primary breeding goal for producing African oil palm genotypes suitable for these subtropical regions.

Microsatellites (simple sequence repeats, SSRs) are tandem DNA repeats of 1–6 nucleotides per unit, and are mostly found in non-coding regions of eukaryotic genomes. Due to low selection pressure in non-coding regions, non-coding SSRs are often highly polymorphic as well as co-dominant and simple to detect. Non-coding SSRs have been widely used for the analysis of genetic diversity and population structure, construction of linkage maps, and detection of quantitative trait loci [2-5]. However, SSRs located in coding and untranslated regions (transcribed SSRs) can be efficient functional markers in genic regions [6]. SSR variation in coding regions can lead directly to functional protein changes, while SSRs occurring in 5′ untranslated regions (5′-UTRs) can affect transcription and translation, and SSRs in 3′-UTRs can affect splicing [7]. Thus, SSRs from transcribed sequences may be directly related to phenotypic variation, and hence functional trait markers.

Molecular markers as AFLPs, RAPDs and AFLPs have been widely used for analyzing genetic diversity and population structure, identification of trait-associated markers and genotype characterization in *Elaeis guineensis* [8-11]. Recently, there is increasing interest in the use of transcriptome sequencing to understand the molecular mechanisms which govern important agronomic traits in *Elaeis guineensis* [12]. Thus, a large number of expressed sequence tags (ESTs) were released. Obviously, this sequence information comprises a valuable resource for identifying gene-associated SSR markers in *Elaeis guineensis*. Previously, EST-SSRs in *Elaeis guineensis* based on this released data have been provided by three studies. Of these three studies, Low et al. [13] reported identification of 648 EST-SSRs associated with tissue culture, while two other studies reported EST-SSRs which were not associated with particular agronomic traits [14,15].

Here, we reported our work on development and characterization of EST-SSR derived from expressed sequences up- or down-regulated at least two-fold in response to cold stress. Our study comprises five parts: (1) Characterization of the frequency and distribution of putative SSRs obtained from *Elaeis guineensis* transcriptome data, (2) analysis of polymorphism in the EST-SSR markers derived from expressed sequences up- or down-regulated at least two-fold in response to cold stress, (3) *in-silico* mapping of these polymorphic markers, (4) assessment of physical distance between these polymorphic markers and candidate genes associated with cold stress, and (5) exploring the population structure of the 192 oil palm lines using the SSR markers linked to candidate genes associated with cold stress. These SSR markers developed in the study will be useful for establishment of genetic mapping as well as population genetic studies, and will provide candidate markers for genetic improvement of cold stress in *Elaeis guineensis*.

## Methods

### Plant materials

The oil palm varieties, *dura* (the thick-shelled African oil palm) and *pisifera* (the thin-shelled African oil palm)*,* were introduced from Malaysia to China in the 1990s and subsequently mutual crossed to produce a large number of F_1_ hybrids. The plantation trial showed that a few F_1_ hybrids can adapt to winter low temperature of Hainan province located on Southern China. The selected F1 hybrid seedlings were treated as follows: F_1_ hybrid seedlings were grown in nurseries. Twenty one one-year-old F1 hybrid plants germinated in the same week and grown in the same nursery were selected for subsequently cold treatment. Prior to cold treatment, the hybrid seedling were placed in a growth chamber at 26°C for one day. Subsequently, spear leaf samples were collected from three individual replicates (as controls) for RNA extraction. The remaining six groups of three seedling replicates were kept at 8°C for 0.5 hours, 1 hour, 4 hours, 8 hours, 1 day and 7 days respectively before sampling. Spear leaves were sampled from control and cold-treated seedlings and immediately frozen in liquid nitrogen. Total RNA was extracted from the control and cold treatment samples based on the MRIP method described by Xiao et al. [16]. mRNA mixtures from the control sample and the cold-treatment sample were prepared in equal proportions for Illumina sequencing.

Moreover, 192 oil palm lines were collected from Hainan province located in Southern China (44) and from Malaysia (148). Among these oil palm individuals collected from Malaysia, 34 were produced by self-pollination of the selected F_1_ plants, showing adaptation to the low winter temperatures in the Hainan province. The other 114 oil palm individuals were recently introduced into China, of which 29 were also produced from the self-pollination of F_1_ plants between *dura* and *pisifera* and for which the pedigrees of the remaining lines were unknown. DNA samples were prepared from young leaves of the 192 oil palm trees using the mini-CTAB methold [17].

### Illumina sequencing and de novo assembly

Purified mRNA isolated from the control sample and from the cold-treatment mixture were separately fragmented with divalent cations under increased temperature. These short fragments were taken as templates to synthesize the first-strand cDNA using hexamer primers and superscript™III (Invitrogen™, Carlsbad, CA, USA). Second-strand cDNA was then synthesized in a solution containing buffer, dNTP, RNaseH and DNA polymerase I and subsequently purified using a QiaQuick PCR extraction kit (Qiagen). EB buffer was used to resolve these short fragments for end reparation and poly (A) addition. The sequence adaptors were linked to two ends of short cDNA sequences and suitably sized cDNA fragments were selected out for PCR amplification based on the agrose gel electrophoresis results. Finally, the library established was sequenced using an Illumina Hiseq™ 2000. The paired-end library was developed according to the paired-End sample Preparation Kit protocol (Illumina, USA). The transcriptome short reads were *de novo* assembled software following the protocol documented by Grabherr et al. [18].

#### Functional annotation of transcriptome data

The transcript sequences were aligned with the NR database at a E-value threshold of 10^−5^ (E-value < 0.00001). Subsequently, the transcript sequences were aligned by BLASTX to protein database, including Swiss-Prot, KEGG and COG. If alignment results of different databases conflicted, BLAST results from NR rather than Swiss-prot were given precedence. The WEGO software was applied to perform GO functional classification of the transcriptome [19]. The result of the GO annotation were also used for KEGG and COG analysis.

#### Calculation of gene differential expression

RPKM (Reads per kb per Million reads) was used to calculate gene expression level. The statistical significance of the differential expression was determined according to the method documented by Audic and Claverie [20]. When thousands of hypothesis tests are performed, the *p*-value suitable for a single test is not sufficient to guarantee a low rate of false discovery. Thus, an FDR (False Discovery Rate) control method was applied using multiple hypothesis testing to correct the *p*-value results [21]. Subsequently, the RPKM ratio was used to compute the fold change of gene expression for each pair of samples simultaneously. The differentially expressed genes were selected using a threshold of FDR ≤ 0.001 and an absolute value of log_2_ ratio ≥ 1 [22].

### Identification of putative SSRs and primer design

The software Msatfinder was used to identify putative SSRs based on the cut-off criteria of 12, 8, 5, 5, 5 and 5 repeats for mono-, di-, tri-, tetra-, penta- and hexa-ucleotide motifs, respectively (http://www.bioinformatics.org/ftp/pub/msatfinder/). Subsequently, primers flanking SSRs were designed using Primer 3 software [23]. Using the software, a total of 3952 primer pairs were designed for these SSR sequences (information listed in Additional file 1). In order to evaluate polymorphisms in SSRs associated with response to cold stress, primers flanking SSRs in expressed sequences that were induced or repressed by low temperatures were used to amplify DNA isolated from the 24 F_2_ oil palm plants.

### PCR amplification and electrophoresis

PCR amplification were performed in 10-μl reaction mixtures containing 100 ng genomic DNA, 10 × PCR buffer, 25 mMMgCl_2_, 1 U TaqDNA polymerase (TaKaRa, China), 0.5 μM of each primer and 0.2 mM dNTP mix, with the following program: denaturation for 5 minutes at 94°C, 35 cycles of 94°C for 30 seconds, 30 seconds at 54.7°C and 30 seconds at 72°C for elongation, with a final extension of 7 minutes at 72°C. PCR products were electrophoretically separated on 1% polyacrylamide denaturing gels and visualized by silver staining. Product sizes were determined by comparison to a 100 bp DNA ladder.

### The diversity analysis of the designed markers and chromosome location

The polymorphic information content (PIC value) was calculated using a PIC calculator (http://www.liv.ac.uk/~kempsj/pic.html) [24]. Using the BLAST algorithm, the chromosomal locations of the polymorphic markers were determined as follows: firstly, the expressed sequences, used to design primers for the polymorphic marker, were BLASTed against the oil palm contig sequences (BioprojectID: 192219: PRJNA192219 *Elaeis guineensis*); secondly, the chromosomal location of the matched contigs was further determined according to the released genome information of Singh et al. [25].

### Population structure

Bayesian clustering was applied to analyze the population structure of 192 oil palm lines using the software STRUCTURE [26]. Ten independent calculations were performed for *K* value (*K* set from 1 to 11). The length of burn-in time and replication number were both set to 100,000 in each run. The maximum likelihood method was applied to assign every oil palm line to a cluster, and the cut-off probability was set to 0.6. The most probable number of true populations (*K*) was identified by plotting △*K* values of K from 1 to 10 in replicate runs for each K and corresponded to the peak of the △*K* graph.

## Results

### Frequency and distribution of gene-based SSRs in the oil palm transcriptomes in response to cold stress

In our research (data unpublished), a total of 51,452 transcripts with an average length of 703 bp were obtained from oil palm transcriptomes in response to cold stress. These transcriptome data is available in TSA (Transcriptome Shotgun Assembly) database of NCBI website (Submission Number: GBSV00000000). Msatfinder identified 5,791 SSR loci located in 5034 transcript sequences (Additional file 1). Nearly one transcript sequence in 10 (5034/51452) contained at least one SSR locus (Table 1). Among these microsatellites identified based on our cut-off criteria, tri-nucleotide motif types were the most abundant (2821, 48.71%). Mono-nucleotide motifs comprised the next largest proportion (1741, 30.06%), followed by di-nucleotide motifs (1124, 19.41%), with a minority of tetra-nucleotide (73, 1.26%), penta-nucleotide (21, 0.36%) and hexa-nucleotide motifs (11, 0.2%).

**Table 1:** **Characteristics of 5791 SSRs identified based on transcriptome data of** ***Elaeis guineensis***

**Motifs**	**Repeat number**															**Total**	**Average repeat number**	**Average repeat length(bp)**
	**5**	**6**	**7**	**8**	**9**	**10**	**11**	**12**	**13**	**14**	**15**	**16**	**17**	**18**	**>18**			
a/t	-	-	-	-	-	-	-	398	272	232	193	147	83	65	310	1700	15.19	15.19
c/g	-	-	-	-	-	-	-	10	8	5	0	2	4	0	12	41	15.85	15.85
tc/ga	-	-	-	137	131	128	61	3	0	0	0	0	0	0	0	460	9.26	18.52
ct/ag	-	-	-	205	152	127	59	1	0	0	0	0	0	0	1	545	12.79	25.58
at	-	-	-	14	10	5	5	0	0	0	0	0	0	0	0	34	9.03	18.06
ta	-	-	-	7	3	8	1	0	0	0	0	0	0	0	0	19	9.16	18.32
ac/gt	-	-	-	3	5	4	2	2	0	0	0	0	0	0	0	16	9.69	19.38
ca/tg	-	-	-	15	16	6	7	3	0	0	0	0	0	0	0	47	9.21	18.42
cg	-	-	-	0	2	0	0	0	0	0	0	0	0	0	0	2	9	18
gc	-	-	-	0	1	0	0	0	0	0	0	0	0	0	0	1	9	18
gag/ctc	191	95	42	2	0	0	1	0	0	0	0	0	0	0	0	331	5.58	16.74
tgc/gca	73	38	34	0	0	0	0	0	0	0	0	0	0	0	0	145	5.73	17.19
cag/ctg	63	44	39	2	0	0	0	0	0	0	0	0	0	0	0	148	5.86	17.58
cgg/ccg	127	66	34	7	0	0	0	0	0	0	0	0	0	0	0	234	9.22	27.66
aag/ctt	74	36	22	1	0	0	0	0	0	0	0	0	0	0	0	133	5.62	16.86
ggt/acc	36	21	7	1	0	0	0	0	0	0	0	0	0	0	0	65	5.58	16.74
ggc/gcc	120	59	35	3	0	0	0	0	0	0	0	0	0	0	0	217	5.66	16.98
gat/atc	25	17	3	0	0	0	0	0	0	0	0	0	0	0	0	45	5.51	16.53
tct/aga	73	43	34	2	1	0	0	0	0	0	0	0	0	0	1	154	5.91	17.73
tga/tca	58	18	9	2	0	0	0	0	0	0	0	0	0	0	0	87	5.48	16.44
gac/gtc	19	6	4	1	0	0	0	0	1	0	0	0	0	0	0	31	5.81	17.43
tcc/gga	151	69	39	3	0	0	0	1	1	0	0	0	0	0	0	264	5.62	16.86
gaa/ttc	86	27	19	4	0	0	0	0	0	0	0	0	0	0	0	136	5.57	16.71
cct/agg	122	69	38	5	0	0	0	0	0	0	0	0	0	0	0	234	5.69	17.07
att/aat	10	10	2	1	0	0	0	0	0	0	0	0	0	0	0	23	5.74	17.22
aac/gtt	7	4	0	2	0	0	0	0	0	0	0	0	0	0	0	13	5.77	17.31
cgc/gcg	61	39	22	3	0	1	0	0	0	0	0	0	0	0	0	126	5.77	17.31
ttg/caa	12	11	2	0	0	0	0	0	0	0	0	0	0	0	0	25	5.6	16.8
agc/gct	40	23	31	2	0	0	0	0	0	0	0	0	0	0	0	96	5.95	17.85
atg/cat	24	10	10	1	0	0	0	0	0	0	0	0	0	0	0	45	5.73	17.19
tta/taa	13	5	2	0	0	0	0	0	0	0	0	0	0	0	0	20	5.45	16.35
cac/gtg	52	9	15	3	0	0	0	0	0	0	0	0	0	0	0	79	5.61	16.83
tgg/cca	61	25	13	3	0	0	0	0	0	0	0	0	0	0	0	102	5.59	16.77
ata/tat	8	1	3	0	0	0	0	0	0	0	0	0	0	0	0	12	5.58	16.74
aca/tgt	7	3	2	1	0	0	0	0	0	0	0	0	0	0	0	13	5.77	17.31
cga/tcg	13	7	3	2	0	0	0	0	0	0	0	0	0	0	0	25	5.76	17.28
cgt/acg	7	4	0	0	1	0	0	0	0	0	0	0	0	0	0	12	5.67	17.01
tag	1	0	0	0	0	0	0	0	0	0	0	0	0	0	0	1	5	15
gta/tac	2	1	0	1	0	0	0	0	0	0	0	0	0	0	0	4	6	18
agt	1	0	0	0	0	0	0	0	0	0	0	0	0	0	0	1	5	15
tetra-	60	12	0	0	0	1	0	0	0	0	0	0	0	0	0	73	5.23	20.92
penta	20	1	0	0	0	0	0	0	0	0	0	0	0	0	0	21	5.05	25.25
Hexa-	8	1	0	0	1	0	1	0	0	0	0	0	0	0	0	11	6	36
Total	1625	774	464	433	323	280	137	418	282	237	193	149	87	65	324	5791	9.21	16.86

**Figure 1:**
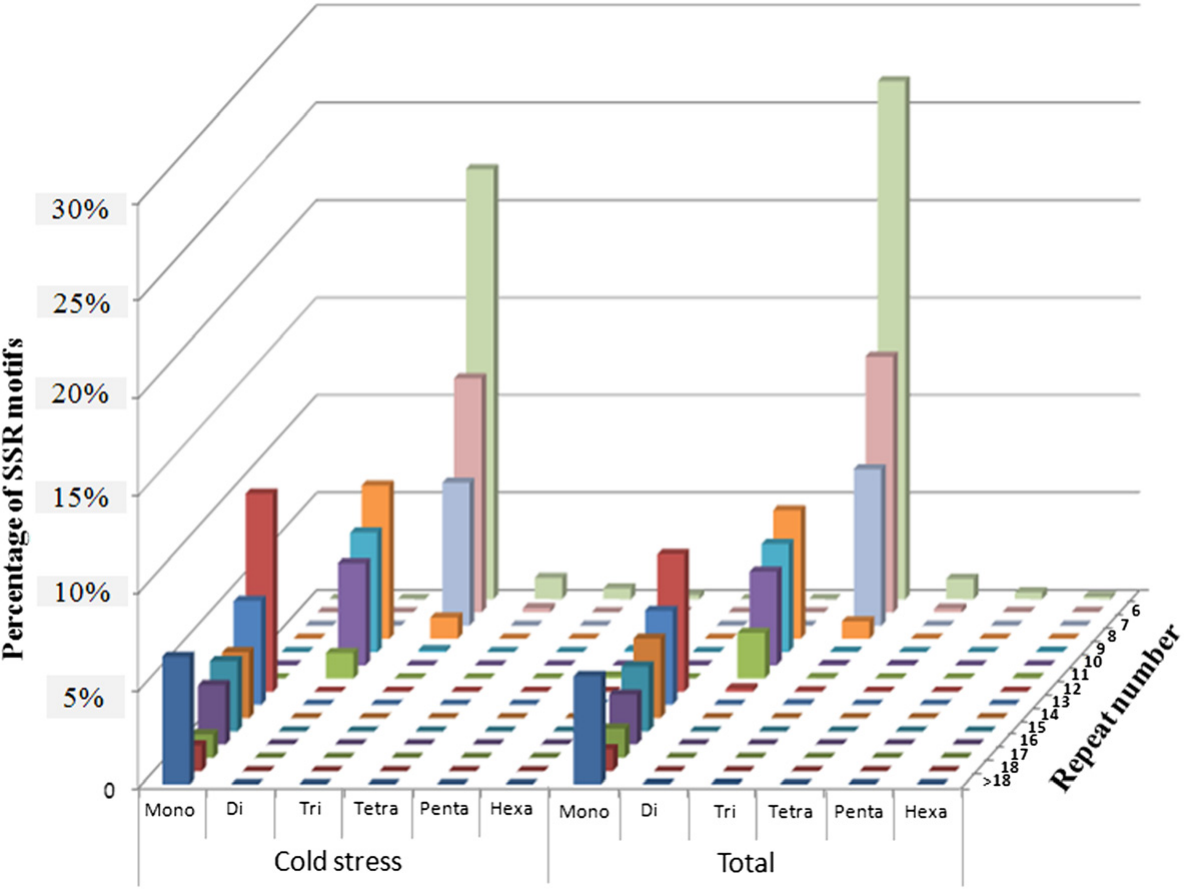
**The distribution of the motif repeats of mono to hexa-nucleotide microsatellites based on all transcript sequences and transcript sequences differentially expressed in response to cold treatment.**

Of the 51,452 transcripts, 10,973 were up-regulated or down-regulated at least two-fold in response to cold stress. The 10,973 transcripts contained 916 identified SSR loci. Identical distribution with respect to microsatellite motif type was observed between all SSR loci identified in the 51,452 transcripts and the 916 SSR loci associated with response to cold stress (Figure 1). Of the SSR loci associated with response to cold stress, tri-nucleotide motif types were the most abundant (42.58%), followed by mono-nucleotide (34.61%) and di-nucleotide (20.52%) motif types.

**Figure 2:**
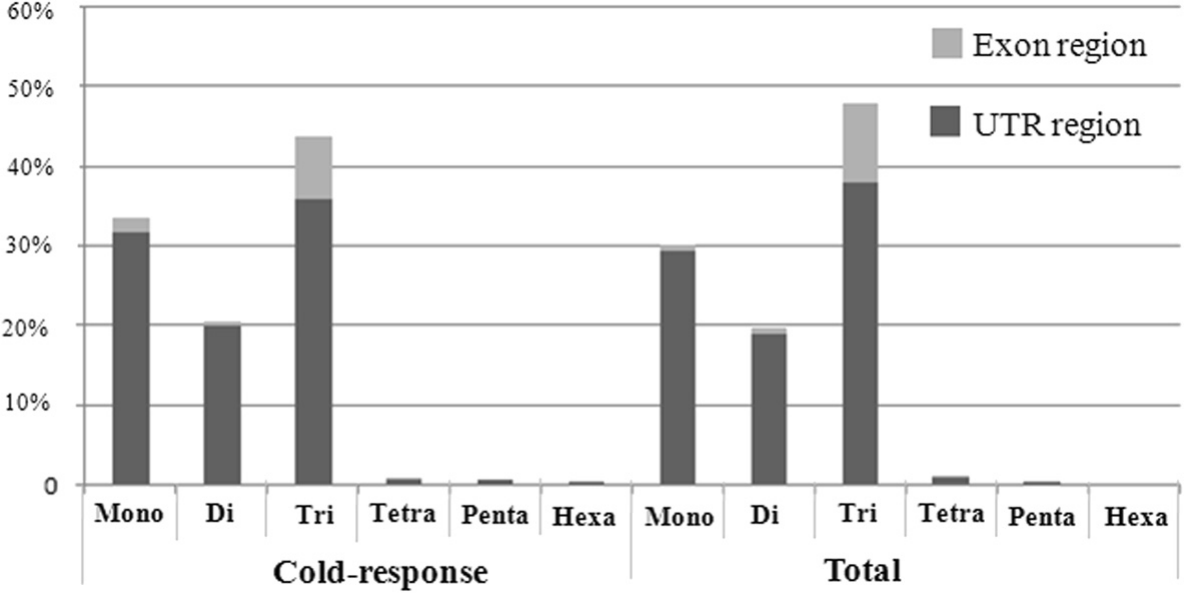
**The percentage distribution of mono-, di-, tri-, tetra-, penta- and hexa-nucleotide repeat SSRs between UTRs and exon regions for total and cold-response-associated SSRs in African oil palm.**

Comparative analysis was performed to ascertain the position within the transcript sequences of both the total SSRs and the cold-response SSRs (Figure 2). Total SSRs and cold-response SSRs both occurred mainly in UTR regions. Of the total SSRs, 1570 mono- repeats (accounting for 90. 02% of the total mono-nucleotides), 1020 di-repeats (accounting for 90.75% of the total di-nucleotides), 2033 tri-repeats (accounting for 79.26% of the total tri-nucleotides), 63 tetra-repeats (accounting for 91.3% of the total tri-nucleotides), 21 penta-repeats (accounting for 100% of the total penta-nucleotides), and 11 hexa- repeats (accounting for 100% of the total hexa-nucleotides) occurred in un-translated regions (UTRs) of expressed transcripts. It should be noted that a largest portion of tri-nucleotide repeats (532, 20.74%) occurred in coding sequences (CDSs) of expressed transcripts. Compared to the total SSRs, the cold-response SSRs showed basically identical distribution within expressed transcripts. However, in cold-response SSRs, a comparative larger proportion of tetra-nucleotide (2, 25%) motif SSRs were located in coding sequences (CDSs) of expressed transcripts.

### Kyoto Encyclopedia of Genes and Genomes (KEGG) pathways of SSR-containing transcripts in response to cold stress

**Figure 3:**
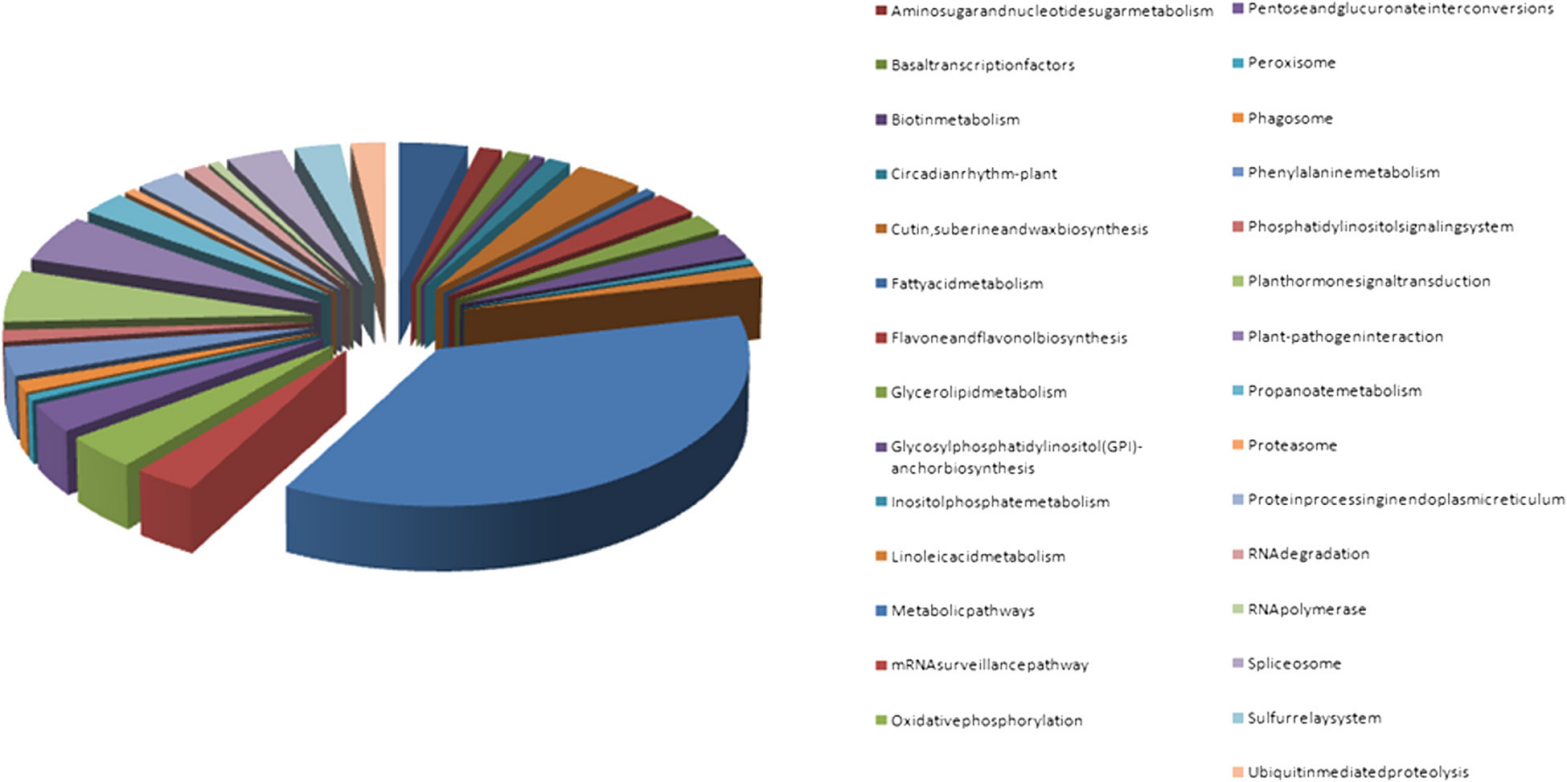
**KEGG annotation of SSR-containing transcripts differentially regulated in response to cold stress in oil palm.**

Annotation of SSR-containing transcripts differentially regulated in response to cold stress showed that these transcripts were unevenly distributed between the different KEGG pathways (Figure 3). Of the 159 SSR-containing transcripts differentially regulated in response to cold stress which could be assigned at least one KEGG pathway, the largest proportion of SSR-containing transcripts (58, 36.48%) were classified into the Metabolic pathways (Pathway ID: ko01100). Plant hormone signal transduction (Pathway ID: ko04075) comprised the next largest proportion (9, 5.66%), followed by plant-pathogen interactions (8, 5.03%; Pathway ID: ko03013), oxidative phosphorylation (6, 3.77%; Pathway ID: ko00190), cutin, suberine and wax biosynthesis (6, 3.77%;Pathway ID: ko00073), and ABC transporters (6, 3.77%;Pathway: ko02010), with single transcripts related to botin metabolism, fatty acid metabolism, inositol phosphate metabolism, peroxisome, proteasome and RNA polymerase.

### Polymorphism in cold-response-associated SSR markers and chromosome positions in *Elaeis guineensis*

**Figure 4:**
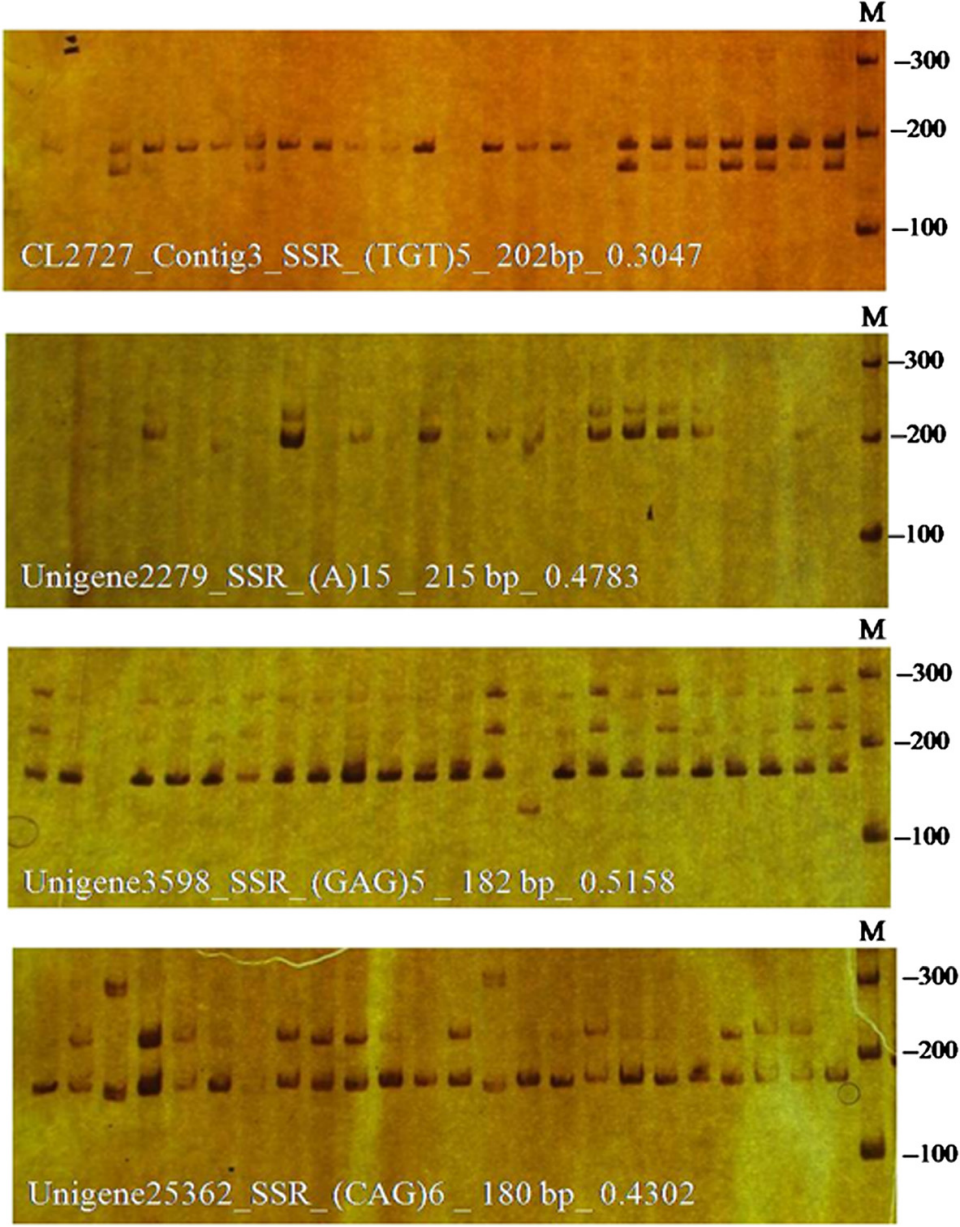
**PCR products and polymorphic characteristics of four SSR markers across 24** ***Elaeis guineensis*** **accessions.**

**Figure 5:**
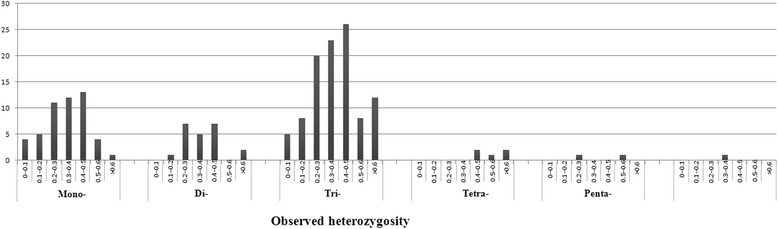
**The distribution of PIC values for mono-, di-, tri-, tetra, penta- and hexa-nucleotide motif SSR loci identified in African oil palm.**

A total of 442 primer pairs were successfully designed from the flanking sequences of cold-response-associated mono- to hexanucleotide SSR repeats. Primer pairs could not be designed for the remaining SSRs, mainly due to difficulties in obtaining sufficient flanking sequences from either side of the identified microsatellites. Subsequently, the 442 pairs of primer sequences flanking 132 mono-nucleotide repeats, 74 di-nucleotide repeats, 219 tri-nucleotide repeats, 7 tetra-nucleotide repeats, 7 penta-nucleotide repeats and 3 hexa-nucleotide repeats were synthesized to test the extent of polymorphism in the cold-response SSRs across the 24 oil palm lines. In 278 (62.9%) of cases, PCR products could be amplified from genomic DNA. The remaining 164 primer pairs were excluded from further analysis due to lack of PCR products or due to weak amplification. Ninety-one primer pairs amplified monomorphic bands in all lines. In total, 182 (41.2%) polymorphic microsatellite markers were identified (Figure 4), including 50 mono-nucleotide repeats, 22 di-nucleotide repeats, 102 tri-nucleotide repeats, 4 tetra-nucleotide repeats, 2 penta-nucleotide repeats, and 1 hexa-nucleotide repeat. The percentage of polymorphic mono-, di-, tri- and tetra-nucleotide repeats was 38%, 30%, 47% and 57%, respectively. From the 182 loci, 402 microsatellite alleles were identified with an average of 2.2 alleles per locus. Of the 402 alleles, 105 were from mononucleotide motif loci with an average of 2 alleles per locus; 46 were from dinucleotide motif loci with an average of 2 alleles per locus, and 227 were from trinucleotide motif loci with an average of 2.2 alleles per locus. Across the 182 polymorphic markers, PIC values ranged from 0.08 to 0.65 (mean = 0.31 ± 0.12), suggesting the cold-response-associated SSR markers developed had moderate levels of polymorphism (Figure 5). The mean PICs of the 50 mono-nucleotide, 22 di-nucleotide and 102 tri-nucleotide repeats were 0.30, 0.31 and 0.31, respectively. Detailed information for the 182 polymorphic markers is listed in Additional file 1.

**Figure 6:**
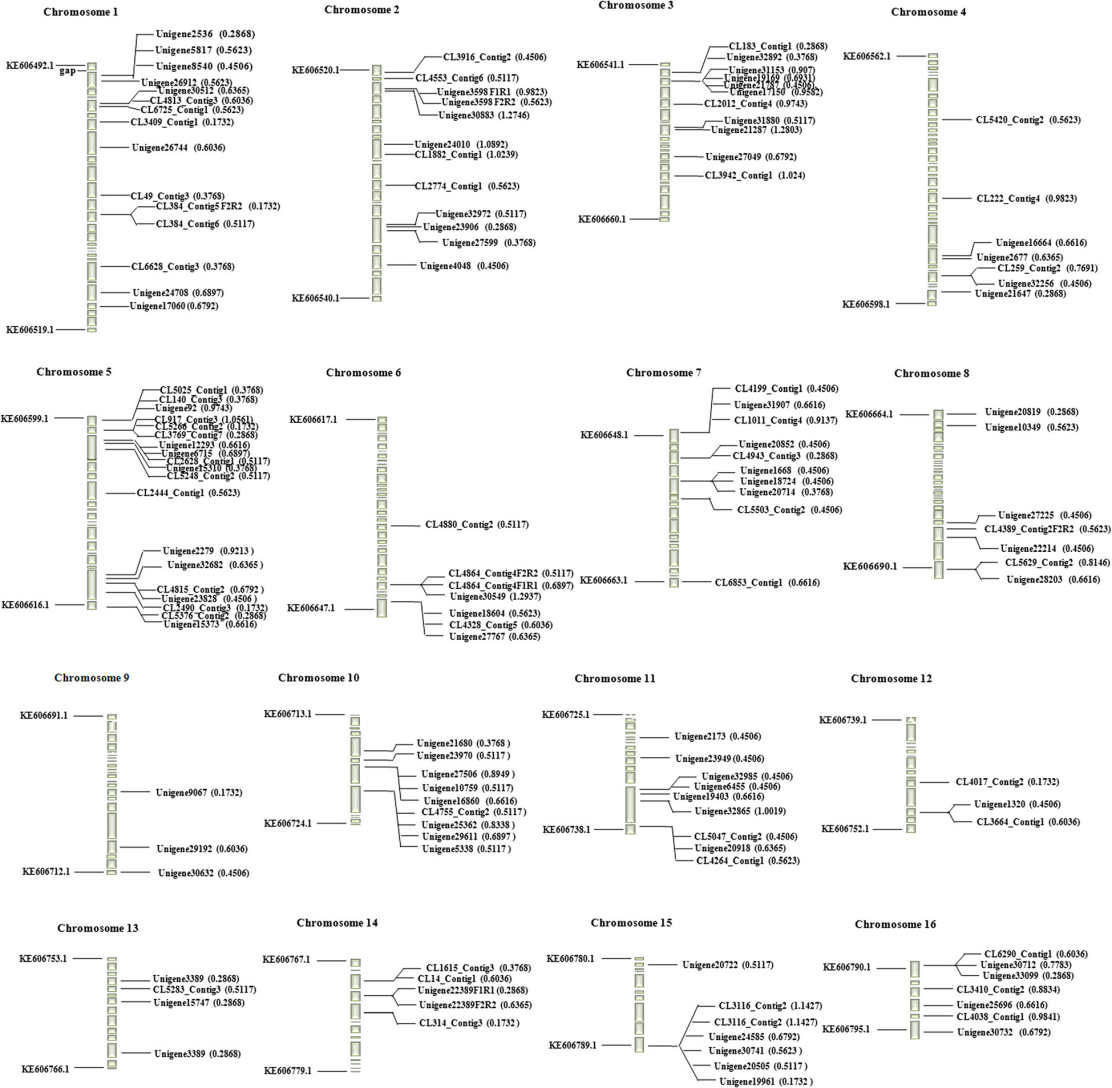
**Chromosomal locations of the gene-based SSRs developed based on transcriptome sequences differentially expressed in response to cold stress.** Chromosomes consist of a series of assembled scaffolds. Every scaffold is represented by a column. The length of the column corresponds to the length of the scaffold: 1 centimeter represents 10000 kb. The left number “KE……” represents the scaffold ID number from the *Elaeis guineensis* genome in the NCBI database. The number in brackets indicates the observed heterozygosity of the SSR markers.

Based on *in-silico* mapping, 137 (75.3%) of the 182 developed gene-based SSR markers could be placed on *Elaeis guineensis* chromosomes (Figure 6). The number of SSR markers per chromosome varied from 3 (chromosome 9) to 20 (chromosome 5), with an average of 8.52 SSR markers per chromosome across the 16 chromosomes. The physical distance between adjacent SSR markers ranged from 96 bp to 20.8 Mbp, with a total coverage length of 473.4 Mbp and an average physical length of 3.5 Mbp. Detailed information for the physical distance between adjacent markers had been listed in Additional file 2.

### Identificaiton of candidate genes in response to cold stress and physical distance between these candidate genes and the SSR markers

The comparative analysis of transcriptomes under cold stress revealed that 10,973 transcripts were up-regulated or down-regulated at least two-fold in response to cold stress. Among these transcripts in response to cold stress, some were functional annotated as cold-resistance genes documented in the previous researches. Based on annotation results, eight *CBF* orthologs, two *ICE1* orthologs, three *SIZ1* orthologs, two *ZAT10* orthologs, one *HOS1* orthlogs and one *MYB15* orthologs were detected, comprising some crucial transcription factors involved in the CBF-mediated cold signal transduction. Of these, six transcripts (35.3%) were up-regulated at least two fold, including Unigene21287 (*ICE1*, 4.49 fold), CL4558.Contig1 (*CBF*, 6.14 fold), CL4552.Contig2 (*CBF*, 11.08 fold), CL83.Contig2 (*CBF*, 5.44 fold), CL83.Contig3 (*CBF*, 7.1 fold) and Unigene 26961 (*CBF*, 11.9 fold). Interestingly, 5′ untranslated region of candidate Unigene21287 (*ICE1*, 4.49 fold) contained a SSR loci (Unigene21287_SSR) with comparatively high diversity extent (PIC value: 0.619) across the 24 lines of *Elaeis guineensis*. Meanwhile, based on *in-silico* mapping, three of the other five candidate genes involved in CBF-mediated pathway were located on genome scaffolds containing SSR markers. The physical distance between the three candidates and adjacent SSR markers were listed in Additional file 3.

In addition, some transcripts were classified as *NAC* transcription factors according to COG annotation results, of which some members have been documented to be related to cold tolerance in some species. In *Elaeis guineensis*, 19 (41.3%) of 46 *NAC* transcription factors were up-regulated at least two fold under cold stress, with fold changes varying from 2.16 fold (Unigene7160) to 10.32 fold (Unigene22381). Of them, the 5′ untranslated region of CL2628.Contig1 (*NAC*, up-regulated 2.82 fold) also contained one SSR maker with moderate polymorphism (PIC value: 0.275) across the 24 lines of *Elaeis guineensis*. Fourteen of other 18 candidate *NAC* transcription factors were also located on genome scaffolds containing SSR markers. The physical distances between the 15 candidates and the adjacent SSR markers are listed in Additional file 4.

Meanwhile, 36 transcripts were functionally classified as putative cold-induced putative orthologs based on annotation results due to previous documentation of cold-inducible expression in other species. However, in *Elaeis guineensis*, only four (10.8%) of 37 transcripts were up-regulated at least two fold in response to low temperature, including CL3095.Contig2 (cold induced protein, 3.67 fold), CL384.Contig1 (cold induced protein, 2.89 fold), CL2052.Contig2 (cold induced protein, 3.75 fold) and CL559.Contig2 (cold induced protein, 2.54). of the four candidates, three were located on genome scaffolds containing SSR markers. The physical distances between the three candidates and their adjacent SSR markers are listed in Additional file 5.

#### Exploring population structure of 192 oil palm lines using ten SSR markers linked to candidate genes

**Figure 7:**
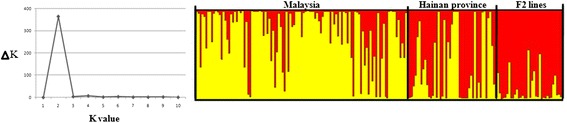
**Population structure of 192 oil palm lines collected from the Hainan province located in Southern China and from Malaysia.**

Ten SSR markers (three closely linked with candidate genes and seven less closely linked to candidate genes, including Unigene21287_SSR, Unigene25696_SSR, CL2628_Contig1_SSR, Unigene19403_SSR, Unigene30741_SSR, CL14_Contig1_SSR, Unigene3598_SSR, CL2490_Contig3_SSR, Unigene32985_SSR, and CL4880_Contig2_SSR) were used to genotype 192 individuals of oil palm collected from Malaysia and China. Of these, 34 lines of oil palm were selected from the F_2_ population derived from self-pollination of the selected F_1_ hybrid that showed adaptation to the low winter temperatures in the Hainan province and 44 were collected from the Hainan province located in Southern China. Other oil palm individuals were recently collected from Malaysia, which did not undergo selection for cold tolerance. The method of Evanno *et al.* [27] was applied to identify the most likely number of ‘true populations’ in the 192 lines of oil palm, two genetic groups were inferred (Figure 7). Structure analysis showed that there is partial separation between these oil palm lines with some cold adaptation and those without. Almost all F_2_ individuals resulting from self-pollination of the selected F_1_ plant were exclusively clustered into the red subgroup (Figure 7). However, oil palm lines collected from Hainan province were found in both subpopulaitons: approximately half (26) were grouped into the red subpopulation. These oil palm lines may be also derived from Southeast Asia and introduced into china in the early twentieth century. Due to lack of adaptation to the climate environment in Hainan province, almost all of the oil palm lines introduced showed low productivity. Subsequently, most of these oil palm lines were cut down and the remaining oil palm lines were used only as aforestation trees. Therefore, there was not extensive artificial selection on the oil palm lines collected from Hainan province, and hence only half can been clustered together with the F_2_ individuals. The majority of the oil palm lines collected from Malaysia were grouped into the yellow subgroup (Figure 7). The oil palm lines were recently introduced into China from Malaysia, and did not undergo the selection for adaptation to the Hainan climatic environment. Therefore, these oil palm lines could grouped into another subpopulation relative to the F2 individuals. In brief, these markers linked to candidate genes can partial distinguish between oil palm adapted or non-adapted to winter low temperatures in the Hainan province, suggesting that these markers may be related to cold stress.

## Discussions

*Elaeis guineensis* has the highest oil yield of any crop species, as well as comprising the richest dietary source of provitamin A [28]. Currently, this crop can only be cultivated in tropical countries. Some effort has been made to introduce *Elaeis guineensis* into subtropical regions worldwide, for example the Yunnan and Hainan provinces in China. However, low winter temperature in these subtropical regions has a serious effect on the flesh fruit productivity of *Elaeis guineensis*. In order to facilitate improvement of cold tolerance in this important crop species, we aimed to develop molecular markers associated with cold tolerance in *Elaeis guineensis*. In this study, we developed 182 polymorphic EST-SSR markers based on sequences differentially expressed in response to cold stress. PIC values of these EST-SSR markers ranged from 0.08 to 0.65 (mean = 0.31 ± 0.12). Meanwhile, based on *in-silico* mapping, the EST-SSR markers were located on each of the 16 *Elaeis guineensis* chromosomes. Subsequently, the physical distances between the developed EST-SSR markers and putative genes related to cold stress were also calculated. Therefore, the EST-SSR markers developed based on sequences differentially expressed in response to cold stress have potential application for association analysis for molecular breeding of cold tolerance in *Elaeis guineensis*.

In previous studies, EST-SSRs were generally identified based on sequencing of *Elaeis guineensis* cDNA libraries. Compared to Illumina sequencing, sequencing of cDNA libraries produces very limited expressed sequence data. Tranbarger et al. [14] identified 465 EST-SSRs from 6,103 non-redundant ESTs derived from cDNA libraries of developing vegetative and reproductive tissues in *Elaeis guineensis*. Of these, only 289 primer pairs flanking the EST-SSRs could be designed. Low et al. [13] identified 648 non-redundant EST-SSRs from 9584 expressed sequence tags in a total of 12 standard cDNA libraries, representing three main developmental stages in oil palm tissue culture. Ting et al. [15] identified 722 SSRs from 10258 unique sequences. In this study, we identified a total of 5,791 SSRs, a considerably greater number than identified in previous studies. Meanwhile, 3952 primer pairs were designed for these SSR sequences, which is far more than the number of SSR pairs developed in the previous studies in *Elaeis guineensis* [13-15]. Of these primer pairs, we focused on 442 primer pairs corresponding to the expressed sequences which were induced or repressed at least two-fold under cold stress.

Based on cut-off criteria of 12, 8, 5, 5, and 5 repeats for mono-, di-, tri-, tetra-, penta- and hexa-nucleotide SSRs, tri-nucleotides were the most abundant EST-SSR markers. This result is identical to previous findings of tri-nucleotide motifs as the most frequent EST-SSR motif in *Cocos nucifera* [29]. However, the most abundant motifs are dinucleotides in some other species [30], which may be a result of loose cut-off criteria to identify SSRs. In order to compare the overall density of SSRs in the *Elaeis guineensis* transcriptome with that reported in other plant species, we re-computed SSRs using the same cut-off criteria as Cardle et al. [31], with 7, 5, 4 and 4 repeats for di-, tri-, tetra- and penta-, respectively. A total of 4794 SSRs were identified with one SSR per 7.53 kb. The SSR density in *Elaeis guineensis* is similar to that in coconut palm (one SSR per 7.59 kb) and soybean (one SSR per 7.4 kb) [29,32], but higher than in maize (one per 8.1 kb), tomato (one per 11.1 kb), Arabidopsis (one per 13.83 kb), poplar (one per 14 kb) and cotton (one per 20 kb). Moreover, higher SSR density than oil palm was found in castor bean (one SSR per 1.77 kb) [33,34].

Some studies have showed that SSRs are mainly located in the UTR regions of expressed sequences, especially in the case of mono-, di-, tetra-, penta-, and hexa-nucleotide motif SSRs [35]. Obviously, if the SSR (for mono-, di-, tetra-, penta-, and hexa-nucleotide motifs) is located in the coding region, mutation in the SSR sequence will cause variation in the coding frame and lead to detrimental mutations. However, a high proportion of tri-nucleotide motifs were found within the coding regions of coconut and castor bean, which may be due to the fact that copy number mutations in tri-nucleotide motifs cannot lead to frame shift mutation. We also observed a high proportion of mono- (97.09%), di- (97.05%), tetra- (91.3%), penta- (100%) and hexa-nucleotide (100%) SSRs in UTR regions in this study. However, for tri-nucleotide motif SSRs, only 20.7% were located in coding regions, much less than in coconut palm (53.6%) [29] and castor bean (76.1%) [35]. The low frequency of SSRs occurring in coding regions may indicate that coding regions are less variable and prone to mutation in *Elaeis guineensis*.

Identification of putative SSRs based on available expressed sequences from the NCBI databases has previously been carried out. However, the extent of polymorphism in these putative SSRs was not described in previous research [13-15]. In our study, 3952 primer pairs flanking the corresponding expressed sequences were designed based on cold-responsive transcripts of *Elaeis guineensis*. Of the 3952 primer pairs, 442 primer pairs flanking the expressed sequences differentially regulated in response to cold stress were used to genotype 24 lines of *Elaeis guineensis*. A total of 182 SSR loci were polymorphic and their PIC value ranged from 0.08 to 0.65, with an average of 0.31. The cold-responsive SSR markers developed in our study seem to have relatively similar levels of diversity to EST-SSRs reported in other species [35,36]. However, the diversity of these SSR markers was lower than previously documented genomic SSRs in other palm species [37]. This can be explained by the fact that SSRs obtained from expressed sequences undergo selection pressure against mutation due to their presence in functional genes.

Although a large number of studies have reported the development of EST-SSR markers in various plant species, chromosomal locations of these developed SSR markers is generally lacking, which is disadvantageous for subsequent studies of linkage disequilibrium, association analysis and molecular breeding. In our study, 137 (75.3%) of the 182 markers developed were located onto the 16 chromosomes of *Elaeis guineensis* based on *in-silico* mapping, which will provide basic information for subsequent genetics and breeding studies. Moreover, plant response to low temperature is a very complex biological process, which requires integration of a large number of genes functioning together to defend against cold stress. The CBF cascade has been documented to have an important role in cold acclimation in diverse plant species. The CBF cascade involves a series of transcription factors, including *ICE1*, *HOS1*, *MYB15*, *SIZ1* and *ZAT10*, transmitting cold signals and subsequently initiating immediate responses to cold stress [38]. In the present study, eight *CBF* orthologs, two *ICE1* orthologs, three *SIZ1* orthologs, two *ZAT10* orthologs, one *HOS1* orthlogs and one *MYB15* orthologs were also detected in *Elaeis guineensis* transcriptomes in response to low temperatures. Just like in other species, putative *ICE1* and *CBF* orthologs were also strongly induced when *Elaeis guineensis* suffered cold stress. Two SSR makers were closely linked separately with an *ICE1* candidate (Unigene21287, up-regulated 4.49 fold) and an *CBF* candidate (CL83.Contig3, 7.1 fold) respectively: one SSR marker was located in 5′ untranslated region of the *ICE1* candidate and another SSR markers was only 12.6 kb from the *CBF* candidate. The two candidate SSR makers should have immediate application for molecular breeding of cold tolerance in *Elaeis guineensis*. Meanwhile, much evidence had also revealed that *NAC* genes also play important roles in abiotic and biotic stress responses [39]. In the study, 46 putative *NAC* orthologs were predicted, of which 19 were up-regulated at least two fold. Among *NAC* candidates induced by low temperature, one SSR markers seemed to be closely linked to two *NAC* candidates (CL4107.Contig2 and CL2628.Contig1): located in the 5′ untranslated region of the *NAC* candidate (CL4107.Contig2) and only have 79.8 kb physical distance away from CBF candidate. This SSR marker closely linked to candidate genes induced by cold stress can be further validated for subsequently association analysis in *Elaeis guineensis*.

In addition, ten SSR markers (three closely linked with candidate genes and seven less closely linked to candidate genes) were used to analyze the population structure of 192 oil palm lines. Interesting, these markers could partial distinguish between oil palm lines that had historically undergone adaptation to climatic environment of Hainan province and those that had not. However, the structure results did not conclusively confirm the relationship between these markers and cold stress. In future, phenotypic data for the cold tolerance of the 192 oil palm lines will be investigated. Association analysis between phenotypic variations for cold stress and the SSR markers linked to candidate genes could further validate if these markers are predictive of cold tolerance in oil palm.

## Conclusions

Gene-based SSRs can directly influence phenotype and also be in close proximity to genetic variation in coding or regulatory regions corresponding to traits of interest. In the study, a total of 5,791 SSR loci were identified based transcriptome data of *Elaeis guineensis* separately from a control (control growth condition) RNA sample and a mixed RNA sample with cold treatment. Of these 5791 gene-based SSRs, 916 were derived from expressed sequences up- or down-regulated at least two-fold in response to cold stress. Based on the flanking sequence of the cold-reponsive SSRs, 442 primer pairs were designed and subsequently used to genotype 24 lines of *Elaeis guineensis*. The PCR amplification products of 182 primer pairs showed polymorphism between the 24 lines. These polymorphic markers were subsequently used for analysis of genetic diversity and population structure, identification of trait-associated markers and genotype characterization in *Elaeis guineensis*. Meanwhile, 137 of these SSR markers were mapped onto the 16 different chromosomes of *Elaeis guineensis* using in-silico mapping, which will provide basic information for location of important agronomic traits and the analysis of linkage disequibrium in *Elaeis guineensis*. Moreover, differential expression analysis showed that one *ICE1* putative ortholog, five *CBF* putative orthologs, 19 *NAC* transcription factors and four cold-induced orhologs were up-regulated at least two fold in response to cold stress. Among these, 22 candidates could be *in-silico* mapped on to genome scaffold containing SSR markers, of which three SSR markers were closely linked with an *ICE1* candidate, a *CBF* candidate and two *NAC* candidates. These three candidate SSR makers would have immediate application for molecular breeding of cold tolerance in *Elaeis guineensis*.

## Additional files

Additional file 1:
**The information of 3952 SSR primers, including primer sequence, Tm value, fragment size, motif, repeat number, Tm (annealing temperature), fold change, na*(Observed allele number), PIC (Polymorphic Information content) and genebank accessions.**
Additional file 2:
**Physical distance between adjacent markers located on the same chromosome.**
Additional file 3:
**Physical distance between markers and candidate genes involving in CBF-mediated pathway.**
Additional file 4:
**Physical distance between SSR markers and candidate genes of NAC family.**
Additional file 5:
**Physical distance between SSR markers and candidate genes involving in cold inducible and cold shock.**
